# Institute of Dental Pedagogics

**Published:** 1903-12

**Authors:** 


### INSTITUTE OF DENTAL PEDAGOGICS.

THE meeting of the Institute this year will be held at Buffalo, beginning December 28 and continuing three days.

The programme, as presented upon another page, indicates an unusually interesting meeting and with subjects attractive to all dental college men. Buffalo is, in many respects, an ideal place for a national meeting, being comparatively easy of access from most centres of dental teaching, and the meeting, therefore, should be well attended.

It is a matter of regret that it was ever deemed necessary to have two national dental college organizations, each holding separate meetings yearly and attended by the same men. It is a great waste of energy, time, and money, and means should be taken to unite this work with that of the National Association of Dental Faculties. As stated in a former article, the legislative business of the latter is about completed, and that which is left could be readily relegated to committees, leaving the sessions free for the discussion of educational topics. This would make the meetings of this body attractive and avoid the necessity for a separate organization for this work.

The Institute of Dental Pedagogics has been the means of demonstrating the value of an interchange of views upon the practical as well as the theoretical details of dental procedures, and while it has, perhaps, not materially changed the curriculi of the several dental colleges of the country, it has kept educational thought in a vigorous condition and prevented relapses into obsolete methods, or, in maintaining courses from which progress has been mainly obliterated.

It is hoped, therefore, that if there cannot be a union of the two dental educational bodies, the Institute may continue its good work as a centre of dental thought and experience.

